# Books and Authors

**Published:** 1906-04

**Authors:** 


**A Textbook of Physiology:** for Medical Students and Physicians. By **William H. Howell**, Ph.D., M.D., LL.D., Professor of Physiology, Johns Hopkins University, Baltimore. Octavo volume of 905 pages, fully illustrated. Philadelphia and London: W. B. Saunders & Co., 1905. (Cloth, $4.00 net; Half Morocco, $5.00 net).

It is a genuine pleasure to examine a book that is prepared by so careful an author as is the writer of this one, and by one who realises the responsibilities belonging to a teacher of one of the fundamental sciences. Howell appears in the arena of textbook making for the first time, but he has prepared a work that is likely to stay for a very long period. He understands that in teaching to plastic minds only established facts are to be laid down primarily, and only well defined theories are to be given even secondary importance.

The general plan of the treatise is to be commended, and the experimental work is demonstrated with great clearness. We, furthermore, have been impressed with Howell's dealing with the special senses, and especially with the dioptrics of the eye. The physiology of reproduction is also well handled; indeed, the book from beginning to end is a delight. We are quite sure it is destined to become a leading textbook in the medical colleges. The illustrations are exceptionally good, and the text is printed in as nearly perfect a manner as can be done.

---

**Pathogenic Microorganisms including Bacteria and Protozoa.** A practical manual for students, physicians, and health officers. By **William Hallock Park, M.D.,** Professor of bacteriology and hygiene, University and Bellevue Hospital Medical College, and director of the research laboratory of the New York Department of Health; assisted by **Anna W. Williams,** M.D., assistant director of the research laboratory. Second edition, enlarged, and thoroughly revised; with 165 engravings, and four full-page plates. 800 pp. 556. Lea Brothers and Company, New York and Philadelphia. 1905. (Cloth, $3.75, net).

The author of this manual has attained a reputation as a laboratory teacher second to none, and very justly so, because he is systematic, thorough, and a master of his subject. Bacteriology is a fascinating study, all the more so when the student is guided by such a superior teacher. Park has included the protoza in his manual and as knowledge increases concerning them, their greater importance becomes apparent in the causation of disease.

This edition has undergone complete revision and now represents the most advanced thought concerning pathogenic microorganisms, and, moreover, is especially adapted to the use of both the student and the clinician. The list of bacilli is a long one but Park has not neglected any of importance in his descriptions, hence his book is one of the best yet presented to those who wish to study the subject with which it discourses.

**A Textbook of Practical Therapeutics, with Especial Reference to the Application of Remedial Measures to Disease and their Employment upon a Rational Basis.** By **Hobart Amory Hare,** M.D., B.Sc., Professor of Therapeutics and Materia Medica in the Jefferson Medical College, Philadelphia. With special chapters by **G. E. de Schweinitz, Edward Martin,** and **Barton C. Hirst.** Eleventh edition, enlarged, thoroughly revised, and largely rewritten. Octavo, 910 pages, with 113 engravings and 4 full-page colored plates. Philadelphia and New York: Lea Brothers and Company. 1904. (Price: cloth, $4.00; leather, $5.00; half morocco, $5.50 net).

When a book reaches its eleventh edition there remains very little to say in regard to it. The reviewers have had their innings in dealing with this textbook for fifteen years, and it is found in every library, public and private, worthy the name all over the land and in most foreign countries. Morever, nearly every medical college carries its title on its lists, so that every student and practitioner is familiar with it.

A number of alterations have been made in this edition to meet the requirements of the new pharmacopeia; also a number of other changes have been made to keep pace with the advances which have occurred all along the line of medicine. It is, taken all in all, one of the greatest works of its class in the world of medicine, and is likely to hold its place during the lifetime of its distinguished author, which we hope will be long and happy.

---

**A Manual of Bacteriology.** By **Herbert U. Williams,** M.D., Professor of Pathology and Bacteriology in the Medical Department of the University of Buffalo. Revised by **B. Meade Bolton,** M.D., expert in the Bureau of Amimal Industry, Washington, D. C.. With 108 illustrations. Fourth edition, revised and enlarged. Philadelphia: P. Blakiston's Son & Co. 1905. (Price, Cloth, $1.50).

The popularity of Professor Williams's manual was assured at the outset, because it gave to the student the essentials of laboratory technic in concise form, together with such bacteriological facts as are most necessary to the physician. Recent advances have taken place in this department of medical science, as well as in most others, and Dr. Bolton has brought the book forward to the immediate present. The chapter on immunity is considerably expanded, and many other changes have become necessary. The chapters by Dr. Carpenter and Dr. Clinton are retained and enlarged. The book is in splendid form for both the laboratory worker and the clinician.

---

**A Textbook of Diseases of Women.** By **Barton Cooke Hirst,** M.D., Professor of Obstetrics, University of Pennsylvania. Second edition revised and enlarged. Octavo of 741 pages, with 701 original illustrations, many in colors. Philadelphia and London: W. B. Saunders & Co. 1905. (Cloth, $5.00 net.; sheep or half-morocco, $6.00 net).

This has been termed a companion volume to the author's textbook of obstetrics. Its popularity was attested by the necessity of reprinting it within a few months after the first edition was sent out from the press. The second edition was issued in less than two years from the first, the author purposely delaying it until sufficient time elapsed to enable him to make a thoroughly satisfactory revision.

Hirst has added to his book fifty-seven pages of text and forty-seven new engravings, while thirty old illustrations have been replaced by new ones; hence it has not been revised in a perfunctory manner. The literature of gynecology has been reviewed and thus the present book represents the science and art of the topic as it exists today. Morever, it is so clear in expression, so lucid in description and so accurate in illustration as to make it one of the best textbooks for the student, as well as one of the most valuable reference works for the general practitioner of medicine.

---

**A Textbook of Anatomy.** Edited by **D. J. Cunningham,** F. R. S., M.D., (Edin. and Dubl.), D.Sc., LL.D. (Glasg. and St. And.), D.C.L. (Oxford), Professor of Anatomy, University of Edinburg. Second and thoroughly revised edition. Illustrated with 936 wood engravings from original drawings, many in colors. Imperial Octavo, pp. 1388. New York: William Wood & Co. 1905. (Price, $6.00).

Since Mr. Cunningham's textbook first appeared, nearly four years ago, some changes have been made in the methods of teaching certain portions of anatomy. These have necessitated a few alterations and some additions in this book, though its size and general make-up remain nearly as at first.

As the work now stands it is one of the leading anatomical textbooks of the day, and will be accorded a place in the college lists as such. Mr. Cunningham himself, has gone from Dublin to Edinburgh since he put forth his first edition where he is now professor of anatomy in the University.

To the student of anatomy this treatise is commended for its clearness, completeness, and exactness, while in its detail and illustration it is one of the foremost works.

---

**Berg's Surgical Diagnosis.** A manual of Surgical Diagnosis. For Students and Practitioners. By **Albert A. Berg,** M.D., Adjunct Attending Surgeon to Mt. Sinai Hospital, New York. In one 12mo. volume of 543 pages with 215 engravings and 21 full page plates. Lea Brothers & Co., Publishers, Philadelphia and New York. 1905. (Cloth, $3.25, net).

Not until recently have surgical writers turned their attention to the preparation of works exclusively limited to surgical diagnosis. This manual is prepared by a surgeon of exceptional opportunities for the task he has set for himself. To say that he has done it well expresses in only a half-hearted way the real facts of the case. Not only does the author make clear many of the superficial surgical maladies, but he presents with great clearness the diagnostic features of many deep seated affections of the various cavities. It is an excellent manual for students, and a valuable reminder to the preoccupied general practitioner.

**Manual of Chemistry.** A Guide to Lectures and Laboratory Work for Beginners in Chemistry. A Textbook specially adapted for Students of Medicine, Pharmacy, and Dentistry. By **W. Simon,** Ph.D., M.D., Professor of Chemistry in the College of Physicians and Surgeons, Baltimore, and in the Baltimore College of Dental Surgery, etc. Eighth Edition, revised, with illustrations. Philadelphia and New York: Lea Brothers & Co. 1905. (Price, Cloth, $3.00, net).

We have become accustomed to seeing such frequent editions of this popular manual, that the delay of a year in the appearance of this one was marked. It was caused, however, by the desire of the author to incorporate in it the changes and additions of the new pharmacopeia. It has also been otherwise revised in much detail and represents the present state of the science as it applies to the purposes of this volume.

Many of the chapters on organic chemistry have been rewritten, dental metallurgy has been more fully considered, and many suggestions made by teachers have been complied with. While it is in particular a manual for the student, it is also of special interest to the physician, pharmacist, and dentist; indeed, it is a book that all these several scientific workers can ill afford to do without.

---

**A Manual of Physical Diagnosis, including Diseases of the Thoracic and Abdominal Organs**. For students and Physicians. By **Egbert Le Fevre,** M.D., Professor of Clinical Medicine and Therapeutics in the University and Bellevue Hospital Medical College, Attending Physician to Bellevue Hospital and to St. Luke's Hospital, New York. New (2d) edition, revised and enlarged. In one 12mo volume of 479 pages with 102 engravings and 6 full page plates in black and colors. Lea Brothers & Co., Publishers, Philadelphia and New York. (Cloth, $2.25, net).

Le Fevre's diagnosis took a prominent place among the useful medical books when first published, becoming at once a favorite with practitioners as well as students of medicine. The author is an experienced teacher, hence is familiar with the needs of students, and has constructed a manual that meets the teaching re quired today. It has been revised with care, somewhat enlarged, and improved in its illustrations, until nothing is left to be desired to maintain its position in the front rank of works of its class. We cannot too strongly emphasise the importance of becoming familiar with such a book, and of keeping it at hand for immediate use.

---

**The Principles of Bacteriology.** A Practical Manual for Students and Physicians. By **A. C. Abbott,** M.D., Professor of Hygiene, University of Pennsylvania. Seventh edition, enlarged and thoroughly revised. In one 12mo. volume of 690 pages, with 100 illustrations, of which 24 are colored. Lea Brothers & Co., New York and Philadelphia. 1905. (Cloth, $2.75, net).

Everywhere in its pages this book is teeming with the subject of which it deals. It is a work on bacteriology by a bacteriologist. Since 1891 it has been known to students of bacteriology as well as to physicians interested in the subject, as one of the very best manuals extant; it has kept pace with the progress made in the laboratory through the successive editions put forth—averaging one copy every two years. Infection and immunity have received additional attention in this edition, and many other additions and alterations have been made, making the book in every way give expression to the very best thought and information on the subject of which it treats.

---

**Neurotic Disorders of Childhood.** Including a study of auto- and intestinal intoxications, chance anemia, fever, eclampsia, epilepsy, migraine, chorea, hysteria, asthma, etc. By **B. K. Rachford,** M.D., Professor of Diseases of Children, Medical College of Ohio, Pediatrist to Cincinnati Good Samaritan and Jewish Hospitals. Octavo, pp. 440. NewYork: E. B. Treat & Co. 1905. (Price, $2.75).

The consideration of the disorders dealt with in this book, include some of the most important maladies that afflict young life. The work is divided into two parts, the first consisting, in the main, of a group of papers published in 1893-94 in the Archives of Pediatrics,. revised and brought forward to meet and include the advances that have taken place since they were printed; the second, deals with the individual neuroses.

The author is a pediatrist of experience and writes with a graceful pen, telling what he knows of the topic, and not bothering overmuch as to the opinions of others, except in matters still unsettled. The chapters on enuresis and migraine are of unusual interest, yet the entire book will prove of great service to the junior practitioner who, of necessity, sees many neurotic children.

---

**A Manual of the Practice of Medicine.** By **A. A. Stevens,** A.M., M.D., Professor of Pathology in the Woman's Medical College of Pennsylvania, and Lecturer on Physical Diagnosis at the University of Pennsylvania. Seventh edition, revised. 12mo of 556 pages, illustrated. Philadelphia and London: W. B. Saunders & Co. 1905. (Flexible leather, $2.50, net).

The author of this manual is well known to the professional world as a careful observer and a pains-taking teacher. He has been before the public as an author for fifteen years and his writings command attention. This favorite manual, intended particularly for students, is also useful to physicians as a reminder in hurried moments. It has been revised to meet the demands of the present hour, and is one of the best books of its class at command of students and practitioners of medicine.

---

**Lea's Series of Medical Epitomes.** Edited by **Victor C. Pedersen,** M.D. Practice of Medicine. A Manual for Students and Practitioners. By **Hughes Dayton,** M.D., Principal of the Class in Medicine, New York Hospital, Out-Patient Department. 12 mo., 324 pages. Lea Brothers & Co., Philadelphia, and New York. 1905. ($1.00, net).

A condensation of the essentials of medicine like this becomes of value to students when reviewing for their finals, and to practitioners when they desire to refresh their memories in the hurried moments of an exacting practice. The material has been well chosen and is put together in excellent form. It constitutes one of the important numbers of this attractive series.

---

**A Textbook of Clinical Diagnosis by Laboratory Methods.** For the use of students, practitioners, and laboratory workers. By **L. Napoleon Boston,** A.M., M.D., Associate in Medicine and Director of the Clinical Laboratories, Medico-Chirurgical College, Philadelphia. Second edition, revised and enlarged, with 330 illustrations, 563 pages, octavo. Philadelphia and London: W. B. Saunders and Company. 1905. (Price, Cloth, $4.00, net).

It is gratifying, indeed, to observe that a second edition of this excellent laboratory manual is required, so very soon after the first; it indicates a healthy growth in the methods of instruction inculcated by this book. The changes in this edition as a matter of course, have been few; nevertheless seventeen pages of new material have been added. The subject of cytodiagnosis, the value of which has been attested, receives increased consideration, and numerous other topics have been introduced.

Boston has prepared one of the best textbooks of its kind that has been printed. Our favorable opinion, expressed in considerable detail a year or more ago, is fully justified and emphasised by this early second appearance of the work.

---

**Saunders's Medical Hand Atlases.** Atlas and Epitome of Diseases of the Skin. By **Professor Dr. Franz Mracek,** professor of dermatology in the University of Vienna. Edited with additions by **Henry W. Stelwagon,** M.D., professor of dermatology, Jefferson Medical College, Philadelphia. Second edition, revised and enlarged, with 77 colored lithographic plates, 50 half-tone illustrations, and 272 pages of text. Philadelphia and London: W. B. Saunders and Company. 1905. (Price, Cloth, $4.00, net).

It is important in the study of diseases of the skin, to have at hand as complete a representation of the clinical appearance of the malady under consideration as it is possible to obtain. In this atlas the artist has used color to the best possible advantage, and has brought out the clinical characteristics of each disease with as much perfection as ever has been done. To possess this book is next to manual possession of the patient. The text is concise, clear and instructive.

---

**Disorders of Metabolism and Nutrition.** A series of Monographs. By **Prof. Carl von Noorden,** M.D., Physician-in-Chief to the City Hospital, Frankfort-on-Main. Authorised American edition. Translated under the direction of **Boardman Reed,** M.D. Part 7, Diabetes Mellitus: A Special Course of Lectures. Delivered in the University and Bellevue Hospital Medical College, New York. Small 8vo, 212 pages. E. B. Treat & Co., Publishers, 241-3 West 23d street, New York. (Price, $1.50).

The distinguished author of these lectures limits himself chiefly to the consideration of the pathological chemistry and treatment of diabetes, only here and there touching upon the symtomatology and cause of the disease whenever necessary to clearness.. The complications, too, are only alluded to occasionally, for like reasons. The subject is full of interest and is dealt with by a teacher who has full knowledge of his subject. It is a monograph that will interest every clinician.

